# Cannabis Effects on Neurocognition and HIV-Related Outcomes: Protocol for a Longitudinal Observational Cohort Study

**DOI:** 10.2196/86814

**Published:** 2026-06-08

**Authors:** Isabella Matthews, Allison Wilson, Mainza Durrell, Ella Remund Wiger, Anna Hotton, Joseph E Rower, Ofir Livne, Raul Gonzalez, Dustin T Duncan, Jennifer Manuzak, Phil Schumm, Adam Carrico, Camille Demarco, Ellen Almirol, Leona Kondic, Isaac Toscano, Joseph DeBrosse, Sarah Keedy, John A Schneider, Justin Knox

**Affiliations:** 1 Chicago Center for HIV Elimination, Biological Sciences Division, University of Chicago, 1525 E 55th St. Suite 205, Chicago, IL, 60615, United States; 2 Department of Pharmacology and Toxicology, Center for Human Toxicology, University of Utah, Salt Lake City, UT, United States; 3 Department of Psychiatry, Columbia University Irving Medical Center, New York City, NY, United States; 4 Department of Psychiatry, New York State Psychiatric Institute, New York City, NY, United States; 5 Department of Psychology, Center for Children and Families, Florida International University, Westchester, FL, United States; 6 Mailman School of Public Health, Department of Epidemiology, Columbia University, New York City, NY, United States; 7 Division of Immunology, School of Medicine, Tulane National Primate Research Center, Covington, LA, United States; 8 Department of Microbiology and Immunology, School of Medicine, Tulane University, New Orleans, LA, United States; 9 Center for Translational Data Science, Biologicial Sciences Division, University of Chicago, Chicago, IL, United States; 10 Department of Health Promotion and Disease Prevention, Robert Stempel College of Public Health and Social Work, Florida International University, Miami, FL, United States; 11 Department of Psychiatry and Behavioral Neurosciences, University of Chicago, Chicago, IL, United States; 12 Division of Community Engagement and Implementation Science, New York State Psychiatric Institute, New York City, NY, United States; 13 Department of Sociomedical Sciences, Mailman School of Public Health, Columbia University, New York City, NY, United States

**Keywords:** HIV, cannabis, neurocognition, fMRI, functional magnetic resonance imaging, executive function, risk/reward processing, data triangulation

## Abstract

**Background:**

Heavy cannabis use may impact neurocognitive functions, particularly prefrontal and limbic systems responsible for risk/reward processing and executive function, which are essential for certain health behaviors, such as HIV prevention. Rigorous research into the effects of cannabis on neurocognitive functions remains limited, particularly among populations with a high burden of HIV.

**Objective:**

This study aims to (1) evaluate associations between cannabis use and neurocognition, (2) evaluate associations between cannabis use and engagement in HIV status–neutral care outcomes (eg, pre-exposure prophylaxis persistence, viral suppression), and (3) assess whether cannabis use motivations modify associations between cannabis use and engagement in HIV status–neutral care outcomes.

**Methods:**

This longitudinal cohort study is enrolling a community-based sample of individuals aged 16 to 29 years residing in Chicago using multiple recruitment strategies. Participants complete 3 in-person assessments annually over 2 years that include (1) computer-assisted questionnaires, (2) neurocognitive assessments (functional magnetic resonance imaging, executive function tasks), and (3) biospecimen collection. Triangulation approaches combine objective and self-reported measures.

**Results:**

The Frontal Lobe Outcomes and Well-Being (FLOW) Study was funded by the National Institutes of Health in April 2023, with data collection commencing in October 2023. As of April 2026, 148 participants have been enrolled and completed baseline assessments, with 63 participants completing their first follow-up appointment and 4 participants completing their second follow-up appointment. Following a temporary administrative pause from March to August 2025 and subsequent federal review confirming regulatory compliance, the study resumed operations in July 2025. Recruitment is currently ongoing, with data collection expected to continue through June 2027. Preliminary analyses are pending completion of longitudinal data collection.

**Conclusions:**

This study addresses critical knowledge gaps by examining potential associations between cannabis use, neurocognition, and HIV. While geographic specificity, structural barriers, measurement challenges, and sample size constraints present some limitations, our methodological approaches—including longitudinal design, triangulation of both objective and self-reported measures, and rigorous neurocognitive assessments—strengthen the investigation. Findings will advance understanding of how cannabis use impacts neurocognition and HIV-related health behaviors, potentially informing targeted interventions that address both substance use and HIV transmission.

## Introduction

Cannabis use is increasing in prevalence across the United States, amid a changing legal landscape. Thus far, 24 states have fully legalized recreational cannabis use [1], including Illinois, the site of the current study. While individuals can use cannabis without harm and some report benefits of use [2], emerging evidence suggests potential negative neurocognitive consequences [3-6], particularly during key developmental periods, such as late adolescence and early adulthood [7]. Brain function mediating complex behavior is thought to be particularly vulnerable to cannabis use due to dense endocannabinoid receptor presence in prefrontal and limbic regions. The endocannabinoid system plays a role in neuromaturation and synaptic pruning [8], processes that occur throughout development, and in late adolescence and early adulthood, this process is particularly focused in frontal regions. These developmental processes are important for cognitive functions that guide complex decision-making and behavioral regulation. Two frontal lobe–mediated cognitive functions have particular relevance to health behaviors: (1) risk/reward (R/R) processing and (2) executive function (EF) [9]. R/R involves registering, weighing, and acting on consequential feedback. EF encompasses a range of higher-order cognitive processes, including mental flexibility, working memory, and inhibition. These systems mature throughout emerging adulthood, with reward systems developing earlier than cognitive control systems [10]. EF is essential for complex decision-making [11,12] and for health care engagement and adherence [13-15]. Together, these interconnected systems underlie health decision-making and prevention behaviors during the critical developmental period when cannabis-related alterations may be most impactful.

Heavy cannabis use might alter R/R processing by increasing the likelihood of riskier choices [16], and has shown an association with blunted neural response to negative consequences in frontal regions [17]. Low EF in childhood has been shown to predict engagement in heavy cannabis use later in life [18]. Evidence suggests that heavy cannabis use negatively impacts R/R and EF processes [19] and that blunted frontal neural activation to loss is also associated. However, these have been smaller studies, and not all studies demonstrate such associations [20]. Moreover, there is a dearth of focus on the emerging adult brain. Our team conducted preliminary research to address these gaps and found compelling early evidence regarding associations of cannabis with altered R/R. This was a cross-sectional study that included 33 young adults categorized as engaged in heavy cannabis use in the past year compared to individuals who did not engage in such use. We found that heavy users had altered R/R behavior as well as altered neural activation patterns during R/R, including differential reward sensitivity behaviorally and neural response patterns to win or loss feedback in the ventral striatum and other key R/R brain regions [21].

Cannabis-neurocognition relationships have particular significance for populations with high cannabis use and elevated health risks. Sexual and gender minorities assigned male at birth who are between the ages of 16 and 25 years exemplify this intersection, as they show high cannabis use rates and HIV burden [22-25]. Conflicting evidence exists regarding the positive or negative effects of cannabis use on HIV-related outcomes, with some studies demonstrating that cannabis use is linked with reduced markers of inflammation and immune activation in blood and cerebrospinal fluid [26-28], reduced viral burden and faster HIV DNA decay following seroconversion [29-32], decreased blood-brain barrier permeability [33], and lower central nervous system (CNS) inflammation and neurocognitive impairment [34,35], while other studies have demonstrated higher rates of seroconversion and suboptimal treatment adherence [36,37]. The neurocognitive domains affected by cannabis—R/R processing and EF—may explain these negative associations.

Chicago’s South Side contains the largest contiguous population of this at-risk group in the United States. Therefore, we are conducting the Frontal Lobe Outcomes and Well-Being (FLOW) Study in this community. This is a longitudinal study designed to understand how cannabis use patterns influence neurocognitive functioning and relevant health behaviors. The specific aims of the FLOW Study are as follows:

1. Evaluate associations between cannabis use and neurocognition
2. Evaluate associations between cannabis use and engagement in HIV status–neutral care outcomes (eg, pre-exposure prophylaxis [PrEP] persistence, viral suppression)
3. Assess whether cannabis use motivations modify associations between cannabis use and engagement in HIV status–neutral care outcomes

**Figure 1.**
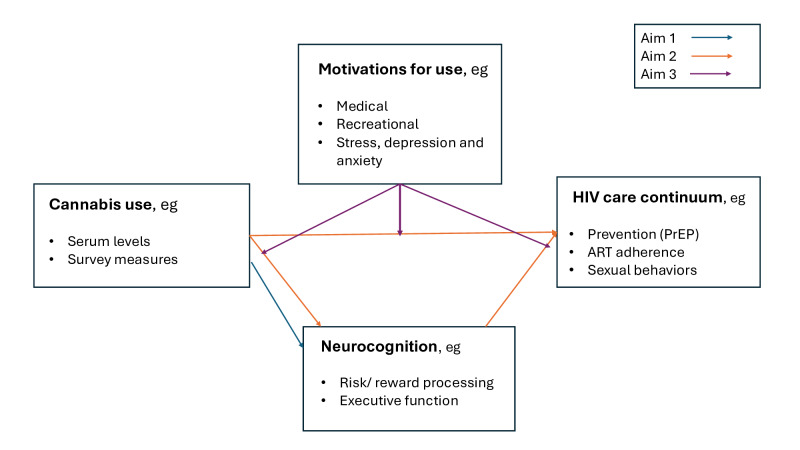
Conceptual model. ART: antiretroviral therapy; PrEP: pre-exposure prophylaxis.

The following conceptual model (Figure 1) provides a visualization.

## Methods

The FLOW Study is a longitudinal observational cohort study. Participants initially complete a baseline session and 2 subsequent follow-up visits every 12 months, totaling 3 visits across a 2-year study period.

### Participants

We are recruiting a convenience sample using multiple recruitment strategies:

1. Distribute flyers with a QR code that opens a text message stating interest in the study, which is then sent to the study team’s phone number. Additional email “blasts” are sent to prospective participants via the study Gmail account.
2. Engage with local high schools, community organizations, and partners who specifically engage with youth and advertise the study at outreach events in these spaces using the flyer with the QR code.
3. Collaborate with the Chicago Center for HIV Elimination (CCHE), an off-campus community service and research space that has served Chicago’s South Side community for over a decade and specializes in HIV prevention and care services [38].
4. Use CCHE’s social media pages to advertise the study.
5. Consult CCHE’s Community Advisory Board for guidance on recruitment strategy and language usage.
6. Enrolled study participants can refer individuals in their social networks to the study using a designated code that they receive upon completion of their first visit. Participants are compensated US $30 via e-payment (eg, Cash App, Venmo, or Zelle) for each person they refer who enrolls in the study, with no limit on the number of referrals.

Interested individuals who contact the study team via these recruitment methods are screened over the phone or in person using a screener survey that is either completed on an iPad or administered by a member of the study team.

### Eligibility Criteria

The following are the inclusion criteria for general participation in the study: (1) assigned male at birth, (2) between 16 and 29 years of age at the time of baseline visit, (3) report attraction to men during their lifetime, (4) able to provide informed consent or assent in English, and (5) reside within Chicago or the metropolitan statistical area and do not plan to move within the next 2 years. This age range was selected to capture participants during active frontal lobe development (aged 16‐25 years) while including older participants (aged 26‐29 years) who serve as a developmental control group, as their brain maturation is largely complete. Exclusion is based on the following criteria: (1) unable to provide informed consent or participate in study procedures; (2) history of significant head injury (loss of consciousness lasting more than 10 minutes and experiencing headache, nausea/vomiting, or dizziness within 24 hours following the incident); (3) serious or chronic health conditions with known impact on neurovascular or neural function, including stroke, epilepsy, brain tumors or cysts, multiple sclerosis, hypertension, diabetes, hydrocephalus, sickle cell disease, autism, blindness, or hearing loss; and (4) history of more than 1 seizure event (seizure events occurring during high fever will be eligible). We will exclude some participants from the functional magnetic resonance imaging (fMRI) only based on the following criteria: (1) contraindications for magnetic resonance imaging (weight greater than 280 pounds, nonremovable ferrous metal in body, claustrophobia, etc) and (2) clinical judgment of being deemed not lucid by the discretion of study staff. This exclusion applies to the EF tests administered as well. In any case of uncertainty, study staff will consult study physicians, who will make judgments regarding suitability for inclusion in the fMRI component.

Notably, this study uses an HIV status–neutral approach, recruiting and serving participants regardless of HIV status to increase inclusivity and minimize stigma. From a neurocognitive perspective, HIV status is unlikely to be a confounding factor, given that participants living with HIV in this age group are expected to represent a smaller proportion of study participants and, while neurocognitive impacts can occur early, most participants have a more recent HIV debut, making HIV-related neurocognitive impacts less severe [39]. All participants receive identical research procedures, and following study visits, participants can access integrated HIV testing, prevention, care, and other health services regardless of their serostatus at CCHE.

### Procedures

Participant assessments are conducted at the University of Chicago Medical Center. The interviewer will first go through the informed consent process, outlining the study, potential risks and benefits, and voluntary nature of participation. For participants who are minors (aged 16‐17 years), a formal assent process will take place prior to participation. A separate, age-appropriate assent form is used to explain the purpose of the study and will be reviewed verbally by trained research staff, with the inclusion of an extra staff member to act as a witness. Participants are also asked to complete a brief self-administered survey measuring contraindications for magnetic resonance imaging scan and cognitive tasks. Afterwards, data are collected through these sources: (1) a computer-assisted participant interview (CAPI) questionnaire, (2) neurocognitive assessments (fMRI, EF tasks), (3) biospecimen collection, (4) extraction of electronic health record data through the use of release of information (ROI) forms, and (5) urine toxicology reports to identify the use of other substances, including marijuana, cocaine or crack, methamphetamine, opiates, and phencyclidine. Detailed outcome measures of the study visit design are listed in Table 1.

**Table 1.** Outcome measures of study visit design.

Timing	Procedures and documentation
Prior to study visit	Interviewer signs and saves informed consent or assent form Apricot check-in (CCHE^a^ research, CCHE attendance, fMRI^b^ participant ID or REDCap^c^ number) Prepare study materials
Day of study visit (with participant)	Identity verification (REDCap form) Informed consent CAPI^d^ assessment Urine toxicology screen; Blood collection (HIV antibody or antigen, cannabis metabolites, and syphilis) Nucleic acid amplification test (chlamydia and gonorrhea) fMRI scan (IGT^e^, MPRAGE^f^, resting state) Neurocognitive testing (WRAT^g^, Trail Making Test, scanning visit log, Verbal Fluency Test, BRIEF^h^, HVLT^i^); distribution of incentives and referral cards
Post visit	Complete REDCap documentation (study incentive form, study visit checklist, State of Mind survey, consenting progress note, successful referral form, if applicable) Send thank-you text message

a CCHE: Chicago Center for HIV Elimination.

b fMRI: functional magnetic resonance imaging.

c REDCap: Research Electronic Data Capture.

d CAPI: computer-assisted participant interview.

e IGT: Iowa Gambling Task.

f MPRAGE: magnetization-prepared rapid gradient echo.

g WRAT: Wide Range Achievement Test.

h BRIEF: Behavior Rating Inventory of Executive Function.

i HVLT: Hopkins Verbal Learning Test.

### Measures

#### Survey

CAPI technology is used to administer the survey. Survey components are primarily administered by study staff, and sensitive components (ie, race-based harassment, posttraumatic stress disorder, and intersectional discrimination) are self-administered by the participants. Survey measures are described in Table 2.

**Table 2.** Measures included in the survey.

Construct and measurement	Reliability (as reported in previous literature)
Demographics (age; height; sexual orientation; ethnicity; relationship status; education; employment; income; incarceration history; housing status; health)	
Sociodemographic questionnaire	N/A^a^
Covariates	
Alcohol use	
Alcohol Use Disorders Identification Test (AUDIT) [40]	0.88
Other substance use	
Questions adapted from the 2020 National Survey on Drug Use and Health (NSDUH) [41]	N/A
Anxiety and depression	
Patient Health Questionnaire-4 (PHQ-4) [42]	0.85
Race-based harassment	
Black Men’s Experiences Scale [43]	0.86
Posttraumatic stress disorder	
PTSD^b^ Checklist for DSM-V^c^ [44]	0.82
Loneliness	
De Jong Gierveld Loneliness Scale [45]	0.89
Intersectional discrimination	
Intersectional Discrimination Scale [46]	0.70‐0.72
Exposures	
Cannabis use	
Daily Sessions, Frequency, Age of Onset, and Quantity of Cannabis Use Inventory [47]	0.69‐0.95
Cannabis Use Disorders Identification Test-Revised (CUDIT-R) [48]	0.73
Marijuana Motives Measure: Cannabis Coping Motives Subscale [49]	0.80‐0.89
Questions adapted from the International Cannabis Policy Study [50]	N/A
Outcomes	
HIV-related behaviors (secondary)	
ART^d^ adherence: Wilson self-report scale [51]	N/A
PrEP^e^ uptake: Questions adapted from the National HIV Behavioral Surveillance System (2013) [52]	N/A
PrEP adherence: Wilson self-report scale [51]	N/A

a N/A: not applicable.

b PTSD: posttraumatic stress disorder.

c DSM-V: Diagnostic and Statistical Manual of Mental Disorders, Fifth Edition.

d ART: antiretroviral therapy.

e PrEP: pre-exposure prophylaxis.

#### Biospecimens

After the survey, trained phlebotomists collect the following biological samples from participants: (1) urine for toxicology screening for substance use; (2) one oral swab and one self-administered rectal swab to screen on site for chlamydia and gonorrhea [53]; (3) two additional self-administered rectal swabs to evaluate levels of proinflammatory cytokines and chemokines; and (4) a blood draw for screening for syphilis and for quantifying cannabis metabolites [54], HIV antibodies, and measures of PrEP usage. The biospecimen collection measures and corresponding constructs are summarized in Table 3.

**Table 3.** Biological data collected at each visit.

Construct	Biologic measure
Substance use	Biologic presence of the following substances in urine samples: marijuana, methamphetamine, cocaine, phencyclidine, and opioids
Sexually transmitted infections	Biologic data for chlamydia, gonorrhea, syphilis in urine, blood, saliva, or rectal mucosa samples
Inflammation	Biologic data on cytokine and chemokine levels in systemic circulation and rectal mucosa samples
Cannabis metabolites	Biologic presence in blood or urine samples
HIV and syphilis	Biologic presence in blood samples

Members of the testing team at CCHE are trained in trauma-informed and youth-centered care to deliver results for HIV and sexually transmitted infections (chlamydia, gonorrhea, and syphilis). Calls are conducted only after confirming the participant’s identity via full name and date of birth and are conducted in a private and confidential space, with no results left on voicemail, via SMS text messaging, or with another individual. Results are provided to participants over the telephone 1 to 2 weeks after their study visit date, with linkage to care options for those seeking treatment or state interest in PrEP. If a participant experiences distress following disclosure of results, immediate support will be provided via a referral for mental health services through CCHE, as well as established organizational safety protocols to ensure participant well-being.

#### Neuroimaging

fMRI scans are completed on a Philips Ingenia 3T scanner at the University of Chicago Magnetic Resonance Imaging Research Center. A scan session is conducted while participants complete the Iowa Gambling Task (IGT), a measure of R/R processing. The IGT [55] is a well-established computerized task designed to assess risk preferences using real-world decision-making. Participants view 4 decks of cards on a screen (labeled as decks A, B, C, and D) and start with a base amount of US $2000. Participants have 100 chances to select a card from the decks and are told, “With each card, you can win some money, but you can also lose some. Some decks will be more profitable than others. Try to choose cards from the most profitable decks so that your total savings will be as high as possible.” When a card is chosen, the subsequent gain or loss of money will appear, marked by red and green status bars at the bottom of the screen. Two decks are coded as being riskier, resulting in an overall loss. The other 2 are coded as safe, with fewer losses and more wins, resulting in an overall gain. Which decks are risky or safe changes between scan sessions. Participants are familiarized with the task before scanning, with a practice session conducted outside the scanner.

Following the IGT, a high-resolution T1-weighted anatomical scan is acquired for alignment and spatial standardization of the functional images across participants. Finally, a 6-minute resting-state scan is conducted with eyes fixated on a white crosshair on a dark background, using similar acquisition parameters as the IGT (gradient-echo echo-planar imaging sequences; repetition time =2 ms; echo time =3.5 ms; 39 3-mm-thick axial slices aligned to the AC-PC line with a 0.6-mm slice gap; 19.2*19.2 cm field of view; SENSE factor =2, flip angle =8°). Four images are acquired and discarded just prior to task start or resting-state sequence start.

#### EF Assessment

Separate from the fMRI scan, EF is measured through multiple tests that sample across the range of processes considered EF. The Behavior Rating Inventory of Executive Function [56] (BRIEF-2 or BRIEF-A [57] for individuals aged 18 years and older or 16 to 17 years, respectively) is considered part of this battery and is administered as part of the survey. Additional EF measures include tests from the NIH Toolbox, which are administered on an iPad. We administer the Flanker Inhibitory Control and Attention Test, List Sorting Working Memory Test, Dimensional Change Card Sort Test, and the Oral Symbol Digit Test. Next, pen-and-paper tests are administered from the Delis-Kaplan Executive Function System [58]: the Trail Making and Verbal Fluency Test. The Word Reading subtest from the Wide Range Achievement Test IV is used to estimate general cognitive ability.

### Statistical Analyses

#### Data Management and Minimizing Missing Data

We code, enter, edit, link, and maintain biological data, electronic medical records, and CAPI-administered questionnaires in REDCap (Research Electronic Data Capture) [59]. Each participant is assigned a unique study ID to enable us to link subfiles. Before each participant leaves the site, their data will be reviewed for completion.

#### Data Triangulation

The principal exposure variable is cannabis use, measured objectively via plasma metabolites of cannabis and triangulated with self-reported cannabis use from the survey using validated measures such as the Cannabis Use Disorders Identification Test-Revised [60]. Objective measures of cannabis use are prioritized using previously validated decision rules for combining objective biomarker data with self-reported substance use, which have been used for stimulants [60] and alcohol [61] and are adapted for cannabis. This approach allows us to account for limitations of respective measures, such as inaccuracy of self-report as well as varying rates of metabolization with cannabis use. It also allows us to precisely delineate participants into potentially relevant categories of use.

HIV prevention and care outcomes are comprehensively assessed using multiple objective data sources triangulated with self-reported measures. Through expanded research ROI forms, we access detailed clinical data, including HIV and sexually transmitted infections testing history, diagnoses and treatment; HIV primary care visits; viral load measurements; antiretroviral therapy and PrEP medication records; receipt of prevention services including PrEP, nPEP, and doxycycline postexposure prophylaxis; mental health and substance treatment services; and case management attendance. This robust clinical dataset enables thorough assessment of the entire PrEP care continuum and HIV care engagement, which is triangulated with validated self-report measures from our survey instrument. Consistent with previous research showing strong correlations between self-reported PrEP use and objective clinical measures, our multimethod approach strengthens data validity by addressing the limitations inherent to any single data collection strategy. This methodology allows for precise characterization of both prevention behaviors among HIV-negative participants and care engagement patterns among participants living with HIV. This expanded research ROI was implemented beginning in March 2025, with data collection through this enhanced protocol applying to participants enrolled from that point forward.

#### Polysubstance Use

We will also rigorously account for other substance use, including substance use disorder, as there is increasing recognition of the need to study substance use as it occurs in real-world settings, including polysubstance use [23,62]. It is difficult to determine whether neurocognition and HIV prevention outcomes are a result of cannabis use alone or a combination of cannabis and other substances. However, it is not necessary to definitively prove that neurocognition and HIV prevention outcomes are due solely to cannabis use in order to identify viable targets for biobehavioral interventions to prevent HIV infection in cannabis users. Nonetheless, to isolate the (causal) effects of cannabis use on neurocognition and HIV prevention outcomes, which is our primary objective, we will measure and control for a wide range of confounding (time-varying) covariates, including noncannabis substance use.

#### Neuroimaging Data Processing

The fMRI data collected from the IGT will be processed using Analysis of Functional Neuro Images [63] and will include realignment of the time series, slice-time correction, high-pass filtering, and spatial normalization. Voxelwise statistical maps of responses to task stimuli (deciding to choose a risky deck, deciding to choose a safe deck, processing “win” feedback, processing “loss” feedback) are generated by estimating beta weights (activation levels) for each condition using an idealized response function. Standard atlas-based masks (Harvard-Oxford) are used to extract mean activation from bilateral R/R brain regions, including the anterior cingulate cortex, medial prefrontal cortex, orbitofrontal cortex, ventral striatum, thalamus, and parahippocampus during win or loss and risky or safe choices. IGT behavioral performance is calculated by subtracting the number of cards picked from safe decks from risky decks. These measures comprise the R/R processing construct.

#### EF Measures

EF assessment includes multiple performance-based and self-report measures. Performance-based measures include NIH Toolbox tests (Flanker Inhibitory Control, List Sorting Working Memory, Dimensional Change Card Sort Test, and Oral Symbol Digit Test), Delis-Kaplan Executive Function System tests (Trail Making Test and Verbal Fluency), and Wide Range Achievement Test IV Word Reading subtest. Self-report measures include the BRIEF-A and BRIEF-2 (for participants aged 16‐17 years). Standard scores are calculated and adjusted for age and education level per manual instructions. These measures comprise the EF construct.

#### Analytic Plan

Separate latent variable models will be developed for R/R processing and EF constructs, allowing for domain-specific analysis of cannabis effects on neurocognitive functioning.

The purpose of these analyses will be to determine both the cross-sectional and longitudinal associations between cannabis use and neurocognitive biomarkers. Let *z*_*ijk*_ and *λ*_*ij*_ be observed measures of cannabis use and neurocognitive performance, respectively, for the *i*th participant at the *j*th time point (*j*=1, 2, or 3). Since multiple measures are being collected for each construct, the subscript *k* is used to identify a specific measure. We will begin by developing an appropriate generalized measurement model for each construct of the form:


(1) $$g_{k}\left\{E\left(z_{ijk}\right)\right\}=a_{k}\left(\theta_{ij}-b_{k}\right),$$

where *θ*_*ij*_ is the true underlying (ie, latent) value of cannabis use for participant *i* at time point *j*. The parameters *a*_*k*_ and *b*_*k*_ capture the discrimination and difficulty of the *k*th measure, respectively. Equation 1 is of the generalized linear model form [64], and a different link function *g*_*k*_(.) is used for each measure to accommodate a mix of measurement types (eg, continuous, binary, ordinal, etc). This approach provides a formal way to combine multiple measures of the same construct (see the Data Triangulationsection above). A similar model will be developed for each neurocognitive construct (eg, R/R and EF); here, we represent the underlying value of neurocognitive performance by *λ*_*ij*_. While this approach is quite flexible, we shall also explore other measurement models, such as those with multiple underlying dimensions (eg, factor analytic models), mixture models with discrete groups (eg, latent class models), or linear or quadratic discriminant analysis.

Next, we will fit longitudinal, mixed-effects models to the underlying values of cannabis use and neurocognitive performance, both unadjusted and adjusted for sociodemographic and other relevant covariates *x*_*i*_:


(2) $$\theta_{ij}=\alpha_{i}+\beta_{1i}t_{j}+\beta_{2}x_{i}+\epsilon_{ij}$$

(3) $$\lambda_{ij}=\gamma_{i}+\eta_{1i}t_{j}+\eta_{2}x_{i}+\epsilon_{ij}$$

where *t*_*j*_=(*j*-1) for *j*=1, 2, or 3. In Equation 2, *α*_*i*_ and *β*_*1i*_ are random effects representing the baseline value of cannabis use and its change over time for the *i*th participant. Likewise, *γ*_*i*_ and *η*_*1i*_ *i*th *β*_*2*_*α*_*i*_*η*_*1i*_*α*_*i*_*γ*_*i*_ represent the association between cannabis use, and *β*_*1i*_*η*_*1i*_ represents the association between *changes* in cannabis use and neurocognitive performance over the 18-month study period. *x*_*i*_*θ*_*ij*_*λ*_*ij*_^4^ may be fit simultaneously using software such as the gsem command in Stata.

The primary purpose of additional analyses is to determine the prospective association between cannabis use at baseline and PrEP care continuum outcomes over the study period, as well as to explore mediation by neurocognition. As described above, we measure these outcomes both objectively and using validated survey measures. Initial analyses model each outcome separately as a function of cannabis use, adjusting for sociodemographic covariates. Generalized linear models are used to accommodate varied measurement types (eg, continuous, binary, ordinal). Since we have multiple outcome measures, naive testing of each increases the likelihood of a type I error—a problem, which we address by combining these into a multivariate outcome model and performing a joint test of the association with cannabis use across outcomes. In addition, we examine whether associations among outcome measures are adequately described by a latent variable model (eg, factor analytic or latent class model). If so, examining the association between the underlying latent variable (measuring PrEP care) and cannabis use provides a simpler description and more powerful test of the association, as well as another way to address the issue of multiple testing. After estimating the overall association between cannabis use and PrEP care, we add measures of neurocognitive performance (RR and EF) to the models. Since several of these models are nonlinear (eg, logistic regression), we use the counterfactual approach to mediation analysis to obtain appropriate estimates of the direct and indirect effects (through neurocognitive performance) of cannabis use on PrEP care [65,66]. This approach is not only applicable to nonlinear models and to situations where there are multiple mediators, but also to cases where there is an interaction between the covariate and the mediator (as would be the case if, for example, those with heavy use *and* poor neurocognitive function were especially likely to have worse PrEP outcomes). Since these outcomes are determined using data extracted from electronic medical records, we expect to have little missing data except for participants who move during the period or are otherwise untraceable. If necessary, we use multiple imputation [67] to address potential bias due to missing data.

Additional analyses are also conducted to determine whether self-reported motivations for using cannabis modify associations between cannabis use, neurocognition, and HIV prevention outcomes. The analyses are conducted by introducing interaction terms between actual cannabis use and motivations for use to generalized linear models predicting PrEP care and HIV transmission behaviors. Two approaches are used. First, in cases where motivation is measured with a single variable, we add and test a single interaction between this variable and cannabis use; this provides the simplest and most powerful test for a moderating effect (assuming linearity). Second, we divide the sample into subgroups of participants with homogeneous motivations and fit a hierarchical model in which the association between cannabis use and PrEP care is allowed to vary across groups. This second approach is more flexible in accommodating multifaceted motivations, and the hierarchical model will help to provide some regularization to avoid overfitting.

#### Power Calculation

Calculations are conducted across a range of scenarios to identify minimum effect sizes detectable with 80% power for binary and continuous outcomes for the primary aims of quantifying associations between cannabis use, neurocognitive outcomes (aim 1), and HIV care engagement (aim 2), and to identify modifiers (ie, interactions) of these associations (aim 3). Because a significantly larger sample size is required to detect statistically significant interaction effects unless the interaction is of very large magnitude, analyses involving effect modification will be considered exploratory. However, we present minimum detectable interaction effect sizes for our given sample size. The estimates presented represent minimum detectable effect sizes estimated under simple, conservative assumptions about total analytic sample size, correlation between repeated measures, and dropout. In practice, we will make use of all available data at all time points, so actual power may be higher. The planned recruitment of 280 participants at baseline and anticipated retention of 206 participants at 2 years (all receiving scans and neurocognitive testing) provide good power for estimating both the cross-sectional and longitudinal associations between cannabis use and neural response. For example, at baseline, 280 participants provide approximately 80% power to detect a correlation of only 0.17 between cannabis use and neurocognitive performance. Conservatively assuming that we will have estimates of change (ie, *β*_1i_ and *η*_1i_) for only those 206 retained at 2 years, we still have approximately 80% power to detect a correlation between changes in cannabis use and neurocognitive performance of 0.19. These calculations assume 2-sided tests at the .05 significance level. As noted above, we expect to retain approximately 80% of the participants to the third time point. To determine whether nonrandom dropout affects our estimates of change in neurocognitive performance, we jointly model the change in neurocognitive performance and the likelihood of attrition [68]. This approach allows us to determine whether the rate of change in neurocognitive performance is associated with the likelihood of attrition and, if so, to adjust our estimate of the mean change accordingly.

For additional analyses looking at prospective associations between cannabis use at baseline and PrEP care continuum outcomes, the proposed sample size provides adequate power to detect small to moderately sized effects. For example, we have approximately 80% power to detect a correlation of only 0.17 between cannabis use and total PrEP time and an odds ratio of 0.66 for a binary outcome such as any PrEP use. We also have reasonable power to detect an indirect effect of cannabis use through its effect on neurocognitive performance. For example, assuming binary measures of cannabis use and PrEP use with an overall odds ratio of 0.9 and a continuous measure of neurocognitive performance correlated at 0.5 with cannabis use, 280 participants provide approximately 80% power to detect an indirect effect that is 37% of the overall effect on the scale of the linear predictor [69,70].

The power to detect interaction effects is less than that to detect main effects. We base this analysis on baseline values of cannabis use and motivations for use, helping to ensure that all 280 participants can be included. A common problem with performing these types of analyses in moderate sample sizes is that statistically significant interaction effects are overestimated (ie, the “winners curse”). The hierarchical model described above provides partial pooling across subgroups with common motivations, which helps to mitigate this issue [71].

### Ethical Considerations

The FLOW Study protocol has been approved by the institutional review board (IRB) at the University of Chicago (IRB23-0687). All study procedures comply with the principles outlined in the Declaration of Helsinki and federal regulations for the protection of human research participants. Written informed consent is obtained from all participants prior to enrollment, with assent procedures implemented for participants under 18 years of age. Study participants receive direct benefits including confidential results from HIV and sexually transmitted infection (STI) testing at each study visit, as well as referrals to integrated health services including HIV prevention and care, substance use treatment, and mental health support through our partnership with the Chicago Center for HIV Elimination (CCHE). Based on recommendations by the Community Advisory Board, participants receive US $200 compensation in cash at each study assessment.

## Results

The FLOW Study was originally funded by the National Institutes of Health in April 2023. Data collection commenced in October 2023 with an original target enrollment of 280 participants. As of October 2025, 148 participants have been enrolled and completed baseline assessments, with 63 participants completing their first follow-up appointment, and 4 participants completing their second follow-up appointment.

The study experienced a temporary administrative pause from March to August 2025 due to a federal funding review. Following a comprehensive review and determination that the study met all regulatory and scientific standards, funding was reinstated, and the study resumed operations in July 2025. Recruitment activities are currently underway as of October 2025, with data collection expected to continue through June 2027.

To date, preliminary analyses have not yet been conducted as the study focuses on completing enrollment and longitudinal data collection. Results are expected to be published following completion of the 2-year follow-up period.

## Discussion

This study aims to advance understanding of the effects of cannabis use on neurocognitive development during late adolescence and early adulthood, a critical period when frontal brain systems are still maturing, as well as its effects on HIV-related outcomes. Our investigation addresses fundamental questions about how cannabis use patterns influence R/R processing and EF—cognitive domains essential for health decision-making and behavioral regulation—and their contribution to HIV transmission.

### Pilot Study Foundations

Our preliminary findings provide compelling evidence that heavy cannabis use may be associated with measurable and concerning neurocognitive alterations. Behaviorally, heavy cannabis users demonstrated initial risk-taking tendencies during a gambling task, making riskier choices early in the paradigm. However, these individuals retained the capacity for adaptive learning and eventually made choices similar to nonheavy users by the end of the task. This pattern suggests that while cannabis use may compromise initial judgment and reward sensitivity, other aspects of cognition and behavior remain feasibly consistent with learning and change, a finding with important implications for intervention design.

Additionally, neuroimaging analyses during R/R revealed neural system properties associated with heavy cannabis use that align with predictions about cannabis effects on developing brain systems. A primary observation was enhanced ventral striatum activation during win relative to loss feedback. This is consistent with heightened reward sensitivity for heavy users and may potentially explain the attraction to immediate gratification over long-term benefits. There was also decreased hippocampal activation during safe gambling choices among heavy users, suggesting altered memory processing during decision-making involving risk assessment. These preliminary observations in a small sample hint at neural system properties that, if confirmed, offer mechanistic insights into how cannabis use may influence health-related decision-making beyond the laboratory. The observed alterations could explain difficulties in treatment-seeking behaviors, medication adherence, and prevention engagement [19,72], as compromised reward processing and EF may interfere with recognizing long-term health benefits over immediate costs.

These cannabis-neurocognition relationships have particular significance when examined in populations facing elevated health risks. Sexual and gender minorities assigned male at birth represent a critical case study for understanding these mechanisms, given both high cannabis use prevalence [73] and disproportionate HIV burden [24]. The neurocognitive domains affected by cannabis—R/R processing and EF—are precisely those underlying HIV prevention behaviors, treatment adherence, and care engagement [25,74,75]. This population provides an ideal context for examining how cannabis-related neurocognitive effects may influence real-world health outcomes. The intersection of substance use patterns with existing health disparities creates conditions where even subtle cognitive alterations could have amplified public health consequences [3,9]. Understanding these relationships in this specific context can inform both targeted interventions for this community and broader cannabis policy considerations.

### Strengths and Limitations

This study has several limitations. First, the study is being conducted in a single urban area, the Chicago metropolitan statistical area, which may limit generalizability to other contexts, particularly areas with different cannabis regulations and consumption patterns [75,76]. We are studying mechanisms that we expect are generalizable; however, we will consider this as we draw inferences from the results. Second, many members of the sample population face structural challenges, including those related to education, employment, income, health care access, and housing instability [76-79]. These factors may affect both cannabis use patterns and study participation and retention [80]. We are attempting to address this by implementing enhanced protocols that include flexible scheduling, transportation assistance, and community-embedded recruitment strategies to maintain engagement despite these obstacles. Third, accurately assessing cannabis use presents methodological challenges due to variations in product potency and consumption methods [81], which may influence neurocognitive effects. In addition, self-reported behaviors may be subject to recall or social desirability bias. To counter these limitations, we use a triangulation approach for all key constructs. We are also using CAPI techniques for assessments, which include self-administered sections to collect sensitive information, helping to minimize social desirability bias [63]. Fourth, while the sample size (N=280) provides adequate power for primary analyses evaluating associations between cannabis use and key neurocognitive domains and HIV prevention outcomes, it may limit detection of subtle interaction effects or smaller effect sizes in neurocognitive subdomains. We attempt to mitigate this limitation by focusing on established neurocognitive measures with demonstrated sensitivity, prioritizing theoretically driven hypotheses, and leveraging longitudinal data to enhance statistical power through repeated measurement.

These limitations notwithstanding, this study represents a substantial advancement in research to understand the impacts of cannabis use on neurocognition and health behaviors related to HIV care and prevention by using rigorous methodologies and a robust sample size. Furthermore, the FLOW Study is conducted among a population with a heavy burden of HIV that is historically underrepresented in substance use research.

### Conclusion

The FLOW Study represents a significant step forward in understanding cannabis-neurocognition relationships during this critical neurodevelopmental window. The complexity of our anticipated findings—demonstrating both impairments and preserved cognitive functions—calls for nuanced approaches to studying cannabis’s effects on neurodevelopment as well as interventions that address these complexities. This is increasingly important given the rising prevalence of polysubstance use and substantial knowledge gaps regarding long-term impacts on brain maturation.

The comprehensive methodology—integrating neuroimaging, behavioral assessment, biomarker analysis, and real-world health tracking—establishes a robust framework for future substance use research, one that will deepen our understanding of the biological mechanisms that facilitate the relationship between cannabis and neurocognition. The longitudinal design will illuminate temporal relationships between cannabis exposure and cognitive changes, addressing fundamental questions about causality that cross-sectional studies cannot resolve.

By examining young sexual and gender minority individuals assigned male at birth in a high-stakes health context, this work demonstrates how basic neuroscience discoveries can be translated into actionable public health insights. The elevated health risks in this population amplify the significance of even modest neurocognitive effects, providing a sensitive context for detecting meaningful relationships between substance use and health behaviors.

These findings will contribute to evidence-based policy discussions about cannabis regulation, inform clinical practice guidelines for health care providers serving young adults, and guide the development of neurocognitive interventions for substance use treatment. Ultimately, this research bridges fundamental neuroscience with applied public health, offering insights relevant to diverse populations while addressing urgent health disparities.

## Supplementary material

10.2196/86814 Peer Review Report 1 Summary comments from reviewers for the study.

## Abbreviations

BRIEF: Behavior Rating Inventory of Executive Function
CAPI: computer-assisted participant interview
CCHE: Chicago Center for HIV Elimination
EF: executive function
FLOW: Frontal Lobe Outcomes and Well-Being
fMRI: functional magnetic resonance imaging
IGT: Iowa Gambling Task
PrEP: pre-exposure prophylaxis
R/R: risk/reward
REDCap: Research Electronic Data Capture
ROI: release of information

## References

[1] (2024). State medical cannabis laws. Centers for Disease Control and Prevention.

[2] Azcarate PM, Zhang AJ, Keyhani S, Steigerwald S, Ishida JH, Cohen BE (2020). Medical reasons for marijuana use, forms of use, and patient perception of physician attitudes among the US population. J Gen Intern Med.

[3] Volkow ND, Swanson JM, Evins AE (2016). Effects of cannabis use on human behavior, including cognition, motivation, and psychosis: a review. JAMA Psychiatry.

[4] Sofuoglu M, Sugarman DE, Carroll KM (2010). Cognitive function as an emerging treatment target for marijuana addiction. Exp Clin Psychopharmacol.

[5] Williams AR, Hill KP (2020). Care of the patient using cannabis. Ann Intern Med.

[6] Rogeberg O, Elvik R (2016). The effects of cannabis intoxication on motor vehicle collision revisited and revised. Addiction.

[7] Curran HV, Freeman TP, Mokrysz C, Lewis DA, Morgan CJA, Parsons LH (2016). Keep off the grass? Cannabis, cognition and addiction. Nat Rev Neurosci.

[8] Fields E, Morgan A, Sanders RA (2016). The intersection of sociocultural factors and health-related behavior in lesbian, gay, bisexual, and transgender youth: experiences among young Black gay males as an example. Pediatr Clin North Am.

[9] Yanes JA, Riedel MC, Ray KL (2018). Neuroimaging meta-analysis of cannabis use studies reveals convergent functional alterations in brain regions supporting cognitive control and reward processing. J Psychopharmacol.

[10] Murray AL, Zhu X, Mirman JH, Ribeaud D, Eisner M (2021). An evaluation of dual systems theories of adolescent delinquency in a normative longitudinal cohort study of youth. J Youth Adolesc.

[11] Swami S (2013). Executive functions and decision making: a managerial review. IIMB Manag Rev.

[12] Anderson P (2002). Assessment and development of executive function (EF) during childhood. Child Neuropsychol.

[13] Perez KM, Patel NJ, Lord JH (2017). Executive function in adolescents with type 1 diabetes: relationship to adherence, glycemic control, and psychosocial outcomes. J Pediatr Psychol.

[14] Brock LL, Brock CD, Thiedke CC (2011). Executive function and medical non-adherence: a different perspective. Int J Psychiatry Med.

[15] Garvie PA, Brummel SS, Allison SM (2017). Roles of medication responsibility, executive and adaptive functioning in adherence for children and adolescents with perinatally acquired HIV. Pediatr Infect Dis J.

[16] Whitlow CT, Liguori A, Livengood LB (2004). Long-term heavy marijuana users make costly decisions on a gambling task. Drug Alcohol Depend.

[17] Wesley MJ, Hanlon CA, Porrino LJ (2011). Poor decision-making by chronic marijuana users is associated with decreased functional responsiveness to negative consequences. Psychiatry Res.

[18] Cavalli JM, Cservenka A, Kerr DCR, Tiberio SS, Owen LD (2023). Ratings of executive function as a risk factor for adolescents’ frequent cannabis use: a prospective longitudinal study. Psychol Addict Behav.

[19] Lawn W, Freeman TP, Pope RA (2016). Acute and chronic effects of cannabinoids on effort-related decision-making and reward learning: an evaluation of the cannabis “amotivational” hypotheses. Psychopharmacology (Berl).

[20] Burke CO, Boutouis S, Spence JS, Filbey FM (2025). Residual and enduring effects of cannabis use on cognitive and psychomotor function: a study of adults during unrestricted cannabis use, short-term abstinence, and protracted abstinence. Exp Clin Psychopharmacol.

[21] Keedy S, Knox J, Schneider J Preliminary results for the study on frequent cannabis use, risk-reward processing, and neural response mechanisms among young black sexual minority men vulnerable to HIV infection. https://cpdd.org/wp-content/uploads/2024/08/CPDD24-Program-Book-FINAL.pdf.

[22] Mimiaga MJ, Reisner SL, Grasso C (2013). Substance use among HIV-infected patients engaged in primary care in the United States: findings from the Centers for AIDS Research Network of Integrated Clinical Systems cohort. Am J Public Health.

[23] Moody RL, Chen YT, Schneider JA (2022). Polysubstance use in a community sample of Black cisgender sexual minority men and transgender women in Chicago during initial COVID-19 pandemic peak. Subst Abuse Treat Prev Policy.

[24] Sullivan PS, Knox J, Jones J (2021). Understanding disparities in viral suppression among Black MSM living with HIV in Atlanta Georgia. J Int AIDS Soc.

[25] Knox J, Hwang G, Carrico AW, Duncan DT, Watson RJ, Eaton LA (2022). Daily marijuana use predicts HIV seroconversion among Black men who have sex with men and transgender women in Atlanta, GA. AIDS Behav.

[26] Manuzak JA, Gott TM, Kirkwood JS (2018). Heavy cannabis use associated with reduction in activated and inflammatory immune cell frequencies in antiretroviral therapy-treated human immunodeficiency virus-infected individuals. Clin Infect Dis.

[27] Ellis RJ, Peterson SN, Li Y (2020). Recent cannabis use in HIV is associated with reduced inflammatory markers in CSF and blood. Neurol Neuroimmunol Neuroinflamm.

[28] Okafor CN, Somasunderam A, Lake JE (2024). Cannabis use and biomarkers of inflammation, immune activation, and microbial translocation in persons with HIV. Cannabis Cannabinoid Res.

[29] Liu Z, Julius P, Himwaze CM (2024). Cannabis use associates with reduced proviral burden and inflammatory cytokine in tissues from men with clade C HIV-1 on suppressive antiretroviral therapy. J Infect Dis.

[30] Falcinelli SD, Cooper-Volkheimer AD, Semenova L (2023). Impact of cannabis use on immune cell populations and the viral reservoir in people with HIV on suppressive antiretroviral therapy. J Infect Dis.

[31] Chaillon A, Nakazawa M, Anderson C (2020). Effect of cannabis use on human immunodeficiency virus DNA during suppressive antiretroviral therapy. Clin Infect Dis.

[32] Milloy MJ, Marshall B, Kerr T (2015). High-intensity cannabis use associated with lower plasma human immunodeficiency virus-1 RNA viral load among recently infected people who use injection drugs. Drug Alcohol Rev.

[33] Ellis RJ, Peterson S, Cherner M (2021). Beneficial effects of cannabis on blood-brain barrier function in human immunodeficiency virus. Clin Infect Dis.

[34] Watson CWM, Paolillo EW, Morgan EE (2020). Cannabis exposure is associated with a lower likelihood of neurocognitive impairment in people living with HIV. J Acquir Immune Defic Syndr.

[35] Watson CWM, Campbell LM, Sun-Suslow N (2021). Daily cannabis use is associated with lower CNS inflammation in people with HIV. J Int Neuropsychol Soc.

[36] Knox J, Magana C, Duncan DT (2025). Cannabis use and HIV among Black sexually minoritized men: a systematic review and narrative analysis. AIDS.

[37] Manuzak JA, Granche J, Tassiopoulos K (2023). Cannabis use is associated with decreased antiretroviral therapy adherence among older adults with HIV. Open Forum Infect Dis.

[38] The Chicago Center for HIV Elimination.

[39] Sanford R, Fellows LK, Ances BM, Collins DL (2018). Association of brain structure changes and cognitive function with combination antiretroviral therapy in HIV-positive individuals. JAMA Neurol.

[40] Bohn MJ, Babor TF, Kranzler HR (1995). The Alcohol Use Disorders Identification Test (AUDIT): validation of a screening instrument for use in medical settings. J Stud Alcohol.

[41] (2021). 2020 methodological summary and definitions. Substance Abuse and Mental Health Services Administration.

[42] Kroenke K, Spitzer RL, Williams JBW, Löwe B (2009). An ultra-brief screening scale for anxiety and depression: the PHQ-4. Psychosomatics.

[43] Bowleg L, English D, Del Rio-Gonzalez AM, Burkholder GJ, Teti M, Tschann JM (2016). Measuring the pros and cons of what it means to be a Black man: development and validation of the Black Men’s Experiences Scale (BMES). Psychol Men Masc.

[44] (2013). Diagnostic and Statistical Manual of Mental Disorders.

[45] De Jong Gierveld J, Van Tilburg T (2010). The De Jong Gierveld short scales for emotional and social loneliness: tested on data from 7 countries in the UN generations and gender surveys. Eur J Ageing.

[46] Scheim AI, Bauer GR (2019). The Intersectional Discrimination Index: development and validation of measures of self-reported enacted and anticipated discrimination for intercategorical analysis. Soc Sci Med.

[47] Cuttler C, Spradlin A (2017). Measuring cannabis consumption: psychometric properties of the Daily Sessions, Frequency, Age of Onset, and Quantity of Cannabis Use Inventory (DFAQ-CU). PLoS One.

[48] Adamson SJ, Kay-Lambkin FJ, Baker AL (2010). An improved brief measure of cannabis misuse: the Cannabis Use Disorders Identification Test-Revised (CUDIT-R). Drug Alcohol Depend.

[49] Simons J, Correia CJ, Carey KB, Borsari BE (1998). Validating a five-factor marijuana motives measure: relations with use, problems, and alcohol motives. J Couns Psychol.

[50] International cannabis policy study. David Hammond PhD.

[51] Wilson IB, Lee Y, Michaud J, Fowler FJ, Rogers WH (2016). Validation of a new three-item self-report measure for medication adherence. AIDS Behav.

[52] Centers for Disease Control and Prevention (CDC) (2013). HIV testing and risk behaviors among gay, bisexual, and other men who have sex with men - United States. MMWR Morb Mortal Wkly Rep.

[53] Gaydos CA, Cartwright CP, Colaninno P (2010). Performance of the Abbott RealTime CT/NG for detection of Chlamydia trachomatis and Neisseria gonorrhoeae. J Clin Microbiol.

[54] Anderson DJ, Freeman TS, Caldwell KS, Hoggard LR, Reilly CA, Rower JE (2025). Analysis of seven selected cannabinoids in human plasma highlighting matrix and solution stability assessments. J Anal Toxicol.

[55] Iowa Gambling Task. ScienceDirect.

[56] Delis-Kaplan executive function system. Pearson.

[57] BRIEF®-A (old version). PAR.

[58] REDCap.

[59] Loflin M, Babson K, Browne K, Bonn-Miller M (2018). Assessment of the validity of the CUDIT-R in a subpopulation of cannabis users. Am J Drug Alcohol Abuse.

[60] Winhusen T, Somoza E, Ciraulo DA (2007). A double-blind, placebo-controlled trial of tiagabine for the treatment of cocaine dependence. Drug Alcohol Depend.

[61] Carrico AW, Hunt PW, Emenyonu NI (2015). Unhealthy alcohol use is associated with monocyte activation prior to starting antiretroviral therapy. Alcohol Clin Exp Res.

[62] Compton WM, Valentino RJ, DuPont RL (2021). Polysubstance use in the U.S. opioid crisis. Mol Psychiatry.

[63] Cox RW (1996). AFNI: software for analysis and visualization of functional magnetic resonance neuroimages. Comput Biomed Res.

[64] McCullagh P, Nelder JA (1989). Generalized Linear Models.

[65] Valeri L, Vanderweele TJ (2013). Mediation analysis allowing for exposure-mediator interactions and causal interpretation: theoretical assumptions and implementation with SAS and SPSS macros. Psychol Methods.

[66] VanderWeele TJ, Vansteelandt S (2014). Mediation analysis with multiple mediators. Epidemiol Methods.

[67] White IR, Royston P, Wood AM (2011). Multiple imputation using chained equations: issues and guidance for practice. Stat Med.

[68] Sudell M, Kolamunnage-Dona R, Tudur-Smith C (2016). Joint models for longitudinal and time-to-event data: a review of reporting quality with a view to meta-analysis. BMC Med Res Methodol.

[69] Vittinghoff E, Sen S, McCulloch CE (2009). Sample size calculations for evaluating mediation. Stat Med.

[70] Gelman A, Hill J, Yajima M (2012). Why we (usually) don’t have to worry about multiple comparisons. J Res Educ Eff.

[71] Thames AD, Mahmood Z, Burggren AC, Karimian A, Kuhn TP (2016). Combined effects of HIV and marijuana use on neurocognitive functioning and immune status. AIDS Care.

[72] Morgan E, Khanna AS, Skaathun B (2016). Marijuana use among young Black men who have sex with men and the HIV care continuum: findings from the uConnect cohort. Subst Use Misuse.

[73] Kipp AM, Rebeiro PF, Shepherd BE (2017). Daily marijuana use is associated with missed clinic appointments among HIV-infected persons engaged in HIV care. AIDS Behav.

[74] Voisin DR, Quinn K, Kim DH, Schneider J (2017). A longitudinal analysis of antiretroviral adherence among young Black men who have sex with men. J Adolesc Health.

[75] Hasin D, Walsh C (2021). Trends over time in adult cannabis use: a review of recent findings. Curr Opin Psychol.

[76] Rafei P, Englund A, Lorenzetti V, Elkholy H, Potenza MN, Baldacchino AM (2023). Transcultural aspects of cannabis use: a descriptive overview of cannabis use across cultures. Curr Addict Rep.

[77] (2024). Fast facts: HIV in the US by race and ethnicity. Centers for Disease Control and Prevention (CDC).

[78] (2019). Understanding and addressing the social determinants of health for black LGBTQ people: a way forward for health centers. https://www.lgbtqiahealtheducation.org/wp-content/uploads/2019/06/TFIE-33_SDOHForBlackLGBTPeople_Web.pdf.

[79] Jenness SM, Maloney KM, Smith DK (2019). Addressing gaps in HIV preexposure prophylaxis care to reduce racial disparities in HIV incidence in the United States. Am J Epidemiol.

[80] Scanlon JK, Wofford L, Fair A, Philippi D (2021). Predictors of participation in clinical research. Nurs Res.

[81] Mariani JJ, Brooks D, Haney M, Levin FR (2011). Quantification and comparison of marijuana smoking practices: blunts, joints, and pipes. Drug Alcohol Depend.

