# Supplementary material for: Cannabis Effects on Neurocognition and HIV-Related Outcomes: Protocol for a Longitudinal Observational Cohort Study
Source: JMIR Res Protoc. 2026 Jun 8;15:e86814. doi: 10.2196/86814 (PMC13246460; doi:10.2196/86814)
Supplement: Peer Review Report 1 [file resprot-v15-e86814-s001.pdf]

SUMMARY STATEMENT
(Privileged Communication)

PROGRAM CONTACT:
Guifang Lao
301-443-1061
laog@mail.nih.gov

Release Date: **06/02/2022**
Revised Date:

Application Number: **1 R01 DA057351-01**

Principal Investigators (Listed Alphabetically):

KNOX, JUSTIN
SCHNEIDER, JOHN (Contact)

Applicant Organization: UNIVERSITY OF CHICAGO

Review Group: **ZDA1 TXT-V (05)**
National Institute on Drug Abuse Special Emphasis Panel
High Priority HIV and Substance Use Research (R01 Clinical Trial Optional)
AIDS

Meeting Date: **05/03/2022**
Council: **AUG 2022**
Requested Start: **12/01/2022**
RFA/PA: **DA22-040**
PCC: **MM/GLA**

Project Title: **Heavy cannabis use, neurocognition and PrEP care engagement among young Black sexual minority men**
SRG Action: **Impact Score:43**
Next Steps: **Visit https://grants.nih.gov/grants/next_steps.htm**
Human Subjects: 30-Human subjects involved - Certified, no SRG concerns
Animal Subjects: 10-No live vertebrate animals involved for competing appl.
Gender: 3A-Only men, scientifically acceptable
Minority: 2A-Only minorities, scientifically acceptable
Age: 6A-Children and Adults, scientifically acceptable

Project Year	Direct Costs Requested	Estimated Total Cost
1	587,767	969,816
2	615,343	1,015,317
3	631,379	1,041,776
4	624,965	1,031,193
5	562,910	928,802
TOTAL	3,022,364	4,986,904

1R01DA057351-01 Schneider, John

COMMITTEE BUDGET RECOMMENDATIONS

RESUME AND SUMMARY OF DISCUSSION: This new application entitled “Heavy cannabis use, neurocognition and PrEP care engagement among young Black sexual minority men” is submitted in response to RFA-DA-22-040: High Priority HIV and Substance Use Research (R01 Clinical Trial Optional) by University of Chicago with Dr. John Schneider as the contact Principal Investigator (PI). Dr. Justin Knox, from Research Foundation for Mental Hygiene, is the other PI on this multiple PI application. The application proposes to examine the associations between heavy cannabis use, neurocognition, sex behavior and PrEP care engagement among young Black gay, bisexual and other sexual minority men (YBSMM) in order to identify potential mechanisms to improve HIV prevention.

During the discussion, the panel notes some strengths. The investigative team is well qualified with the appropriate complementary expertise. The application is significant because it focuses on YBSMM, a high-risk population for HIV acquisition with a prevalent use of heavy cannabis as well as low PrEP uptake and persistence. The scientific premise is strong and builds on existing preliminary studies showing adolescent brains may be particularly susceptible to maladaptive changes related to cannabis use and that heavy cannabis use is associated with increased risk of HIV transmission. Rigorous objective measures are used to assess cannabis use, neurocognition, and data triangulation. Administering an fMRI-based neurocognitive measure longitudinally among emerging adults is innovative. The research environments at the associated institutions are excellent.

The application also has some weaknesses. The eligibility criteria for baseline cannabis use are not well defined. The intervention translation group meeting and subsequent focus groups are not well described. The application does not adequately address how social and structural factors drive cannabis use among YBSMM or their connections to HIV risk. Feasibility of conducting multiple MRIs in this cohort is not clearly established. It is not thoroughly detailed how qualitative data will be analyzed and integrated into the study. It is also unclear how the results will yield inferences on HIV and substance use policy. There may be considerable overlap in contributions among the investigators. Furthermore, while Dr. Knox is responsible for technical oversight, his offsite location from the Chicago site may not be ideal for this role.

Based upon the evaluation of scientific and technical merit, this application received an Overall Impact/Priority score of 43.

DESCRIPTION (provided by applicant): We propose to conduct research that will determine the impact of relevant behaviors on HIV prevention among Young Black Sexual Minority Men (YBSMM), such as heavy cannabis use. Data from our group and others demonstrate heavy cannabis use as prevalent and increasing in YBSMM community members, is associated with HIV acquisition, use as a sex-drug, greater likelihood of membership in an HIV transmission cluster, and decreased HIV testing. We propose to explore mechanisms, specifically neurocognitive impacts of heavy cannabis use, linking heavy cannabis use to HIV prevention outcomes, and whether motivations for cannabis use, amidst a changing cannabis regulatory, social acceptance and legal landscape, modify its effects on HIV prevention. In the proposed study, we will rigorously examine links between heavy cannabis use, neurocognition, sex behavior and PrEP care engagement. First, we will elucidate the effects of cannabis use on neurocognition - specifically, brain systems supporting risk/reward (RR) processing, as well as higher order organizational functions collectively referred to as executive function (EF) in YBSMM. Second, we will explore how cannabis use, directly and via neurocognitive impacts, is associated with HIV prevention, and particularly PrEP care engagement (primary outcome). The proposed study will integrate and expand these lines of research within the context of traditional health department and CDC supported HIV prevention programs that engage YBSMM in the South Side of

Chicago and adjacent suburbs, home to the largest contiguous majority Black population in the US. We will use rigorous objective measures to assess cannabis use (e.g., quantification of cannabis metabolites in plasma), neurocognition (e.g., neuroimaging) and PrEP outcomes (e.g., EMR measured persistence), and triangulate that data using validated survey measures. We will also rigorously account for other substance use, as a proportion of YBSMM who use cannabis also use other substances (e.g., alcohol), and there is increasing recognition of the need to study substance use as it occurs in real-world settings, including polysubstance use. We will assess these factors longitudinally over 1.5 years (3 times 9 months apart) in a cohort of 280 YBSMM living without HIV, to permit examination of within-individual biological changes and the dynamic nature of cannabis use and its association with prevention care outcomes. We aim to: 1) Determine cross-sectional and longitudinal associations between cannabis use and neurocognition (i.e., neural response to risk/reward processing and executive function) in a cohort of YBSMM; 2) Evaluate overall, direct and indirect (via neurocognition) associations between cannabis use and PrEP care (e.g., persistence [primary]) and HIV transmission behaviors (e.g., group sex [secondary]); and 3) Determine whether motivations for cannabis use modify associations between cannabis use and HIV prevention outcomes. Identifying neurocognitive mechanisms through which cannabis use affects HIV prevention and the importance of motivations for cannabis use in understanding clinical outcomes will provide targets for future HIV prevention efforts.

PUBLIC HEALTH RELEVANCE: We propose to conduct research that will determine the impact of relevant behaviors on HIV prevention among Young Black Sexual Minority Men (YBSMM), such as heavy cannabis use. We propose to explore mechanisms, specifically neurocognitive impacts of heavy cannabis use, linking heavy cannabis use to HIV prevention outcomes (and particularly PrEP use), and whether motivations for cannabis use, amidst a changing cannabis regulatory, social acceptance and legal landscape, modify its effects on HIV prevention. Identifying neurocognitive mechanisms through which cannabis use affects HIV prevention and the importance of motivations for cannabis use in understanding clinical outcomes will provide targets for future HIV prevention efforts.

CRITIQUE 1

Significance:	2
Investigator(s):	3
Innovation:	2
Approach:	5
Environment:	2

Overall Impact:

The proposed study will investigate mechanisms, specifically neurocognitive impacts of heavy cannabis use, link heavy cannabis use to HIV prevention outcomes-specifically PrEP care engagement, and determine whether motivations for cannabis use, amidst a changing cannabis regulatory, social acceptance and legal landscape modify its effects on HIV prevention. The proposed sample is 280 YBSMM ages 16-25 years in Chicago, Illinois. The study will include rigorous objective measures to assess cannabis use (e.g., quantification of cannabis metabolites in plasma), neurocognition (e.g., neuroimaging-fMRI) and PrEP outcomes (e.g., EMR measured persistence), and triangulate that data using validated survey measures. Factors will be assessed longitudinally over 1.5 years at three time points, each nine months apart. If successful, this research would have a moderate impact on understanding the role of cannabis use in PrEP engagement vis-à-vis neurocognitive mechanisms. The proposed application of this knowledge in tailored “neurofeedback” interventions and substance use and HIV policy is questionable however it may be applied in other ways. The premise for this research is strong. Low uptake of PrEP among sexually active YBSMM is seen as an important barrier to reducing HIV infections in this population. The investigators are well qualified to lead the proposed

research, and their complementary expertise is appropriate although there is considerable overlap among investigators and in current research projects presented as preliminary evidence for this project. The environment is well-suited to support the proposed research. There are a good number of strengths including objective measures, scientific premise, and focus on YBSMM, however there are several weaknesses in the approach which further moderated enthusiasm, specifically eligibility criteria are not well defined, the intervention translation group meeting and subsequent focus groups are underdeveloped, and although mentioned, it remains unclear how social-structural factors driving cannabis use as well as changing social acceptance and legal landscapes will be accounted for in this study.

1. Significance:

Strengths

- Focus on YBSMM, a high-risk population for which PrEP uptake/persistence is low is important to addressing persistent HIV and PrEP inequities.
- Heavy cannabis use is highly prevalent in YBSMM, yet its impact on neurocognition and HIV prevention remains understudied.
- Establishing a greater understanding of how cannabis use may influence PrEP has potential to contribute to the development of more effective interventions to promote PrEP persistence.

Weaknesses

- The suggested future directions of developing tailored neurofeedback interventions and informing substance use and HIV policy may not be realistically acceptable or feasible.
- Lack of focus on salient and dynamic social and structural factors driving cannabis use among YBSMM and their connection to HIV risk, or a justification for why these factors are not adequately included.

2. Investigator(s):

Strengths

- Overall, this is a strong investigator team with the substantive and methodological expertise needed to conduct the proposed study as demonstrated in publications and research studies in overlapping areas.
- Experienced investigators with a track record in recruitment and retention of YBSMM in HIV and substance use related research.
- The team is also enhanced by the inclusion of an existing community advisory board.

Weaknesses

- Roles could be further clarified as there is overlap in the contributions of Dr. Duncan (Co-I), Dr. Schneider (MPI), and Dr. Knox (MPI).
- Dr. Knox's role is not well explained or justified—all study activities are in Chicago.
- While select members of the team have worked together, it is unclear how often the team will meet and troubleshoot project issues—some components indicate biweekly, others monthly.

3. Innovation:

Strengths

- Focus on triangulating objective and self-reported measures of cannabis use with PrEP care engagement will address limitations in prior research.
- Administering an fMRI-based neurocognitive measure longitudinally among emerging adults—a noted neurocognitive developmental period.

Weaknesses

- Using focus groups to gather feedback on investigator-developed intervention strategies is not innovative.

4. Approach:

Strengths

- Objective measures of cannabis use with validated survey measures of self-reported cannabis use.
- Integrating EMR data with self-report PrEP uptake and care engagement as a non-invasive alternative to DBS measurement of PrEP.
- Building on existing preliminary studies conducted by the investigative team establishes the role of cannabis use in HIV risks and potential role in HIV prevention.
- Comprehensive sample recruitment and retention procedures previously used by members of the investigative team to ensure cohort retention.
- The focus on YBSMM, and the heterogeneity that exists among this group to study HIV inequities, is appropriate and well justified.

Weaknesses

- There is a description of acquiring qualitative data from open-ended survey questions and focus groups (post intervention training working group meeting), yet there is no mention of how these data will be analyzed and integrated into the study.
- Eligibility is unclear in terms of baseline cannabis use and how that could potentially affect sampling approach or variability in the cohort.
- Although the team conducted a feasibility study of MRI with the study population, this cross-sectional study does not indicate feasibility of conducting multiple MRI in the cohort.
- Intersectionality and expertise in intersectionality is indicated, but it is unclear how it is used in the study development or aligns with the study measures and analyses.
- Details regarding the study procedures are lacking (e.g., method and mode of administering screening, linkage to care strategies for participants who test positive).
- Intervention training working group is a lost opportunity to have community input in the translation of this research. A three-day retreat for investigators to then share interventions strategies in focus groups is not novel or necessarily translational.
- It is unclear whether the proposed study aims are unique to the other cohort studies used as preliminary evidence for this application, and that the application highlights as having (on the surface) some overlap.
- Unclear from the analyses how the results will yield inferences on HIV and substance use policy—these factors are not measured nor are they integrated into study findings.

5. Environment:

Strengths

- Strong scientific environment at the institutions to which the investigators are members.

Weaknesses

- Referral opportunities related to sexual health and substance use/misuse for participants could be strengthened.

CRITIQUE 2

Significance:	1
Investigator(s):	2
Innovation:	3
Approach:	3
Environment:	1

Overall Impact:

This submission from Schneider and colleagues proposes to conduct an observational cohort study of HIV-negative young black sexual minority men (N=280, 18-month follow-up) to identify associations and mechanisms underlying heavy cannabis use, neurocognition, and PrEP engagement. To accomplish this, the investigators propose Aim 1) to determine cross-sectional and longitudinal relationships between cannabis use and risk/reward processes and executive function (psychometric instruments and fMRI). Aim 2) evaluate direct and indirect (mediated by neurocognition) associations between cannabis use and PrEP engagement (e.g., persistence). Aim 3) determine whether the motivation for heavy cannabis use modifies the association between cannabis use and HIV prevention outcomes.

The proposal has notable strengths including a highly significant area of focus (addressing persistently high HIV seroconversion rates among YBSMM), a successful research team with a track record of work in this field, mechanistic insight from fMRI, inclusion of 16–18-year-old participants, and a nuanced consideration of confounders and use of appropriate statistical approaches. Weaknesses include i) an investigator-heavy team, ii) limited real-world applicability at present, and iii) failure to address how transitions to long-acting PrEP agents over the course of the study will affect inferences. Overall, the strengths outweigh the weakness and leave me with enthusiasm for the proposal.

1. Significance:

Strengths

- YBSMM remain at high risk for and interventions to reduce HIV incidence in this group are significant.
- Heavy cannabis use is highly prevalent among YMSMM but has been less investigated than stimulant and alcohol use in this setting.
- As the legal status of cannabis evolves across the US, cannabis-related problems may increase.
- Preliminary data show that adolescent brains may be particularly susceptible to maladaptive changes related to cannabis use and that heavy cannabis use is associated with increased risk of HIV transmission.

Weaknesses

- The degree to which these research aims can be translated to field interventions, at least at present, is debatable. However, the proposed “Intervention Translation Workshop” in year 5 of the study is an admirable attempt to spark this connection.

2. Investigator(s):

Strengths

- The PI and co-Is have many publications and an established track record in the field of HIV prevention in key populations.
- The team includes expertise on HIV prevention and YBSMM (Schneider, Duncan, Knox), fMRI (Keedy), clinical pharmacology (Rower), neuropsychology (Gonzalez) and statistics (Schumm).

Weaknesses

- The team may be investigator-heavy, with 7 named investigators and overlapping expertise among Schneider, Duncan, and Knox.

3. Innovation:

Strengths

- The focus on PrEP engagement as the target behavior is appropriate, as PrEP is likely to be more effective in preventing HIV transmission than approaches that focus on modifying sexual risk behaviors.
- The investigators' use of fMRI to assess risk/reward processing and executive function as correlates of heavy cannabis use and as a predictor of PrEP persistence is innovative.

Weaknesses

- Similar approaches and methods have been applied previously to stimulant and alcohol use in YBSMM.

4. Approach:

Strengths

- The investigators appear to have carefully considered the many confounding factors (with polysubstance use being among the most important) that must be addressed in the analysis.
- Use of objective measures (serum cannabis metabolites, EMR data for PrEP use) when feasible.
- The inclusion of YBSMM 16 to 18 years of age is important and well-justified as persons in this high-risk age group are often excluded.

Weaknesses

- The application fails to address temporal changes in PrEP that are likely to occur during the study period: transition from daily or on-demand use of oral TFV/FTC to long-acting injectables: cabotegravir injections every 2 months (FDA-approved) and lenacapavir injections every 6 months (phase-3 trials underway).
- It is unclear while why biologic measures of tenofovir exposure are not proposed in PrEP persistence.
- Fentanyl, which now commonly adulterates stimulants and cannabis, is not directly addressed.

5. Environment:

Strengths

- Chicago and UC provide an outstanding environment for the proposed study.

Weaknesses

- None noted.

CRITIQUE 3

Significance:	2
Investigator(s):	2
Innovation:	2
Approach:	2
Environment:	1

Overall Impact:

The proposed study aims to explore neurocognitive impacts of heavy cannabis use, linking heavy cannabis use to HIV prevention outcomes and examine whether motivations for cannabis modify its effects on HIV prevention among YBSMM in Chicago. This is a clear, well-written proposal with the potential for significant impact. The focus on cannabis, particularly given increasing legalization, is of great importance, particularly for Black SMM. This is led by an excellent team of researchers, led by Drs. Schneider and Knox (MPIs). Additional strengths of the study include the longitudinal design, objective measures of cannabis use, nerocognition, and PrEP outcomes. The impact is slightly lowered due to some concerns about the intervention translation workgroup retreat and the influence of structural-level factors. Overall, this is an innovative strong proposal with high potential impact.

1. Significance:

Strengths

- This study is highly significant, particularly given the increasing legalization of cannabis and high levels of use among YSMM.
- The examination of PrEP care is important.

Weaknesses

- The proposal makes reference to intersectional identities but does not adequately consider how those societal positions influence drug use and HIV prevention (e.g., criminal justice involvement) and how that may play into the proposed study. (Minor)
- Data from the study will be useful to understand cannabis use and PrEP, yet the translational aspect of the study is lacking given the minimal details provided around the Intervention Translational Workgroup.

2. Investigator(s):

Strengths

- Drs. Schneider and Knox will serve as MPIs; Schneider is a leading expert in HIV and substance use and Knox is a promising junior faculty member.
- Duncan is a social and spatial epidemiologist and former mentor of Dr. Knox and collaborator of Dr. Schneider.
- Dr. Gonzalez is a psychologist at FIU and directs the substance use neropsychology lab.
- Dr. Keedy is a clinical neuropsychologist at University of Chicago with expertise in MRI.

- Dr. Rower has training in clinical pharmacology and is the associate director of the center for human toxicology at the University of Utah.
- L. Philip Schumm will serve as senior biostatistician.
- Several team members have published together and/or served as mentors to Dr. Knox.

Weaknesses

- Knox is MPI but is not on site in Chicago. He will be responsible for “technical oversight”, but his location is not ideal for this. There is also no travel budget included for Dr. Knox to regularly visit Chicago to engage with the study team.

3. Innovation:

Strengths

- The use of fMRI in the proposed study is innovative.

Weaknesses

- The focus solely on Black SMM is appropriate, yet not innovative.

4. Approach:

Strengths

- Strengths of the approach include the use of objective measures to assess cannabis use, neurocognition, and PrEP outcomes.
- This is a longitudinal study conducted over 1.5 years, which will enhance understandings of longer-term effects.

Weaknesses

- The proposal states an estimated 8% will seroconvert during the study, but they don’t indicate what resources (counseling, referrals, etc.) will be in place for those participants.
- “Heavy” cannabis use is not clearly defined.
- There’s not sufficient information provided about the variables included (e.g., no alphas, no indication of whether they’ve been used with this population before, etc.)
- The intervention translation workgroup retreat holds promise, but it’s unclear who is included and what will occur during these 3 days that will translate these data into intervention avenues. Similarly, the team describes focus groups that will be used to help achieve this goal, yet they do not provide detailed information about the procedures, content, or analysis of these data.
- The study leverages existing research relationships with participants in the target demographic. While this may assist in recruitment efforts and trust, this strategy can also promote research fatigue, feelings of being “overly studied” and the team may want to consider these and similar implications.

5. Environment:

Strengths

- The University of Chicago is an excellent environment to conduct the proposed research.
- Dr. Keedy’s lab at U Chicago is excellent and connected to the imaging center. Dr. Schneider is the Director of the Chicago Center for HIV Elimination.

Weaknesses

- None noted.

THE FOLLOWING RESUME SECTIONS WERE PREPARED BY THE SCIENTIFIC REVIEW OFFICER TO SUMMARIZE THE OUTCOME OF DISCUSSIONS OF THE REVIEW COMMITTEE ON THE FOLLOWING ISSUES:

STUDY TIMELINE: NOT APPLICABLE

PROTECTION OF HUMAN SUBJECTS: ACCEPTABLE

INCLUSION OF WOMEN PLAN: ACCEPTABLE

INCLUSION OF MINORITIES PLAN: ACCEPTABLE

INCLUSION OF INDIVIDUALS ACROSS THE LIFESPAN: ACCEPTABLE

VERTEBRATE ANIMALS: NOT APPLICABLE

BIOHAZARD COMMENT: NOT APPLICABLE

FOREIGN INSTITUTION: NOT APPLICABLE

SELECT AGENTS: NOT APPLICABLE

RESOURCE SHARING PLAN: ACCEPTABLE

AUTHENTICATION OF KEY BIOLOGICAL AND/OR CHEMICAL RESOURCES: NOT APPLICABLE

COMMITTEE BUDGET RECOMMENDATIONS: Recommended budget modifications or possible overlap identified:

- Possible overlap with other studies to which Drs. Knox, Schneider, and Duncan are PIs/MPIs. Specifically, concern about scientific overlap with R01 DA054553 (Cannabis use, PrEP and HIV Transmission Risk Among Black MSM in Chicago), on which Duncan and Knox (Co-Is on the current proposal) are MPIs.
- No funding for site visits (Chicago) for Dr. Knox who is based in New York. The travel is limited to investigators for conferences.

Footnotes for 1 R01 DA057351-01; PI Name: Schneider, John

NIH has modified its policy regarding the receipt of resubmissions (amended applications).See Guide Notice NOT-OD-18-197 at https://grants.nih.gov/grants/guide/notice-files/NOT-OD-18-197.html. The impact/priority score is calculated after discussion of an application by averaging the overall scores (1-9) given by all voting reviewers on the committee and multiplying by 10. The criterion scores are submitted prior to the meeting by the individual reviewers assigned to an application, and are not discussed specifically at the review meeting or calculated into the overall impact score. Some applications also receive a percentile ranking. For details on the review process, see http://grants.nih.gov/grants/peer_review_process.htm#scoring.

MEETING ROSTER

The roster for this review meeting is displayed as an aggregated roster that includes reviewers from multiple DA Special Emphasis Panels Meetings
for the 2022/08 council round.
This roster for DA is available here.
